# Spatial and Temporal Patterns of Locally-Acquired Dengue Transmission in Northern Queensland, Australia, 1993–2012

**DOI:** 10.1371/journal.pone.0092524

**Published:** 2014-04-01

**Authors:** Suchithra Naish, Pat Dale, John S. Mackenzie, John McBride, Kerrie Mengersen, Shilu Tong

**Affiliations:** 1 School of Public Health and Social Work & Institute of Health and Biomedical Innovation, Queensland University of Technology, Kelvin Grove campus, Brisbane, Queensland, Australia; 2 Environmental Futures Centre, Australian Rivers Institute, Griffith School of Environment Griffith University, Brisbane, Queensland, Australia; 3 Australian Biosecurity CRC and Faculty of Health Sciences, Curtin University, Perth, Western Australia, Australia; 4 School of Medicine and Dentistry, James Cook University, Cairns, Queensland, Australia; 5 Mathematical Sciences, Queensland University of Technology, Gardens Point campus, Brisbane, Queensland, Australia; Centers for Disease Control and Prevention, United States of America

## Abstract

**Background:**

Dengue has been a major public health concern in Australia since it re-emerged in Queensland in 1992–1993. We explored spatio-temporal characteristics of locally-acquired dengue cases in northern tropical Queensland, Australia during the period 1993–2012.

**Methods:**

Locally-acquired notified cases of dengue were collected for northern tropical Queensland from 1993 to 2012. Descriptive spatial and temporal analyses were conducted using geographic information system tools and geostatistical techniques.

**Results:**

2,398 locally-acquired dengue cases were recorded in northern tropical Queensland during the study period. The areas affected by the dengue cases exhibited spatial and temporal variation over the study period. Notified cases of dengue occurred more frequently in autumn. Mapping of dengue by statistical local areas (census units) reveals the presence of substantial spatio-temporal variation over time and place. Statistically significant differences in dengue incidence rates among males and females (with more cases in females) (χ^2^ = 15.17, d.f. = 1, p<0.01). Differences were observed among age groups, but these were not statistically significant. There was a significant positive spatial autocorrelation of dengue incidence for the four sub-periods, with the Moran's *I* statistic ranging from 0.011 to 0.463 (p<0.01). Semi-variogram analysis and smoothed maps created from interpolation techniques indicate that the pattern of spatial autocorrelation was not homogeneous across the northern Queensland.

**Conclusions:**

Tropical areas are potential high-risk areas for mosquito-borne diseases such as dengue. This study demonstrated that the locally-acquired dengue cases have exhibited a spatial and temporal variation over the past twenty years in northern tropical Queensland, Australia. Therefore, this study provides an impetus for further investigation of clusters and risk factors in these high-risk areas.

## Introduction

Dengue is emerging and resurging as a worldwide public health problem since the 1950s, affecting more than 110 countries today, and is a leading cause of hospitalisation and morbidity among children in the tropics and subtropics [1]. It is a mosquito-borne viral disease caused by one of four dengue viruses transmitted by *Aedes* mosquitoes. Currently mosquito control is the only available mitigation strategy but *Wolbachia* and the genetic manipulation of mosquitoes to lead to male sterility, may change this in the intermediate future. *Aedes aegypti* is the most common dengue-transmitting mosquito in the state of Queensland in Australia. *Aedes albopictus* (Asian tiger mosquito) another mosquito able to transmit dengue is currently threatening the Australian mainland having been detected on a number of Torres Strait islands [2]. *Ae. aegypti* typically breeds in human-made container habitats such as water storage jars in and around human settlements including those in dense urban areas [3]. *Ae. albopictus* breeds in the same containers as *Ae. aegypti* but also breeds in natural containers in the bush such as tree holes, cut bamboo, banana trees and coconut shells. However, both these mosquitoes breed in fresh waters, but not in swamps or creeks. The recent arrival of the exotic species *Ae. albopictus* is of great concern because, if *Ae. albopictus* colonises the mainland, it could extend to the southern states due to its tolerance of more temperate conditions [2].

In Australia, dengue re-emerged in north Queensland during 1992–1993, after disappearing for about 10 years, i.e., 1981–1991 [4]. Since then, outbreaks and epidemics of dengue, with locally-acquired cases, were reported in Cairns and Port Douglas [4]. These are located in the north-east (urban tropical) coastal regions in Queensland. From January 1993 to June 2012, a total of 2,398 locally-acquired cases were documented in Queensland by Queensland Health. Outbreaks of dengue occur primarily in areas where *Ae. aegypti* mosquitoes are found. Dengue viruses may be introduced into areas by travellers who become infected while visiting other areas of the tropics where dengue commonly exists [5].

Most mosquito-borne diseases exhibit spatial and temporal variations in their distribution [6]. Spatial analyses using geostatistics such as spatial autocorrelations, variograms, interpolations, and temporal analyses using chi-squared statistics and time series models are commonly applied to highlight patterns of disease incidence [7], [8].

Geographic information systems (GIS) play an important role in disease surveillance and control of the mosquito-borne diseases as they assist in the analysis of potential risk factors associated with the disease through the geo-coding processes [8] and facilitate maps that are useful for the identification of spatially and temporally localised areas of potential high-risk populations [7]–[11]. The visualised information represented in different types of maps based on GIS enables simultaneous observation of both the attribute and geographical relationships [12]–[14]. Maps also help policy decision-makers and public health officials to communicate with the public and policy decision-makers about complex information in an easily interpretable format [15], [16].

GIS can provide not only an opportunity to improve our understanding of the distribution patterns of dengue, but can also provide an environmentally and socially informed platform to develop the elements of an early warning system towards control and prevention of dengue [8]. The advancement of geographical information systems (GIS) and spatial statistics has greatly improved the understanding of dengue dynamics, including its dependence on ecological factors. Hence, in this study, we examined the spatial and temporal patterns of locally-acquired dengue transmission in northern Queensland, Australia using GIS tools and geostatistical techniques.

## Methods

### Study area

**Figure 1:**
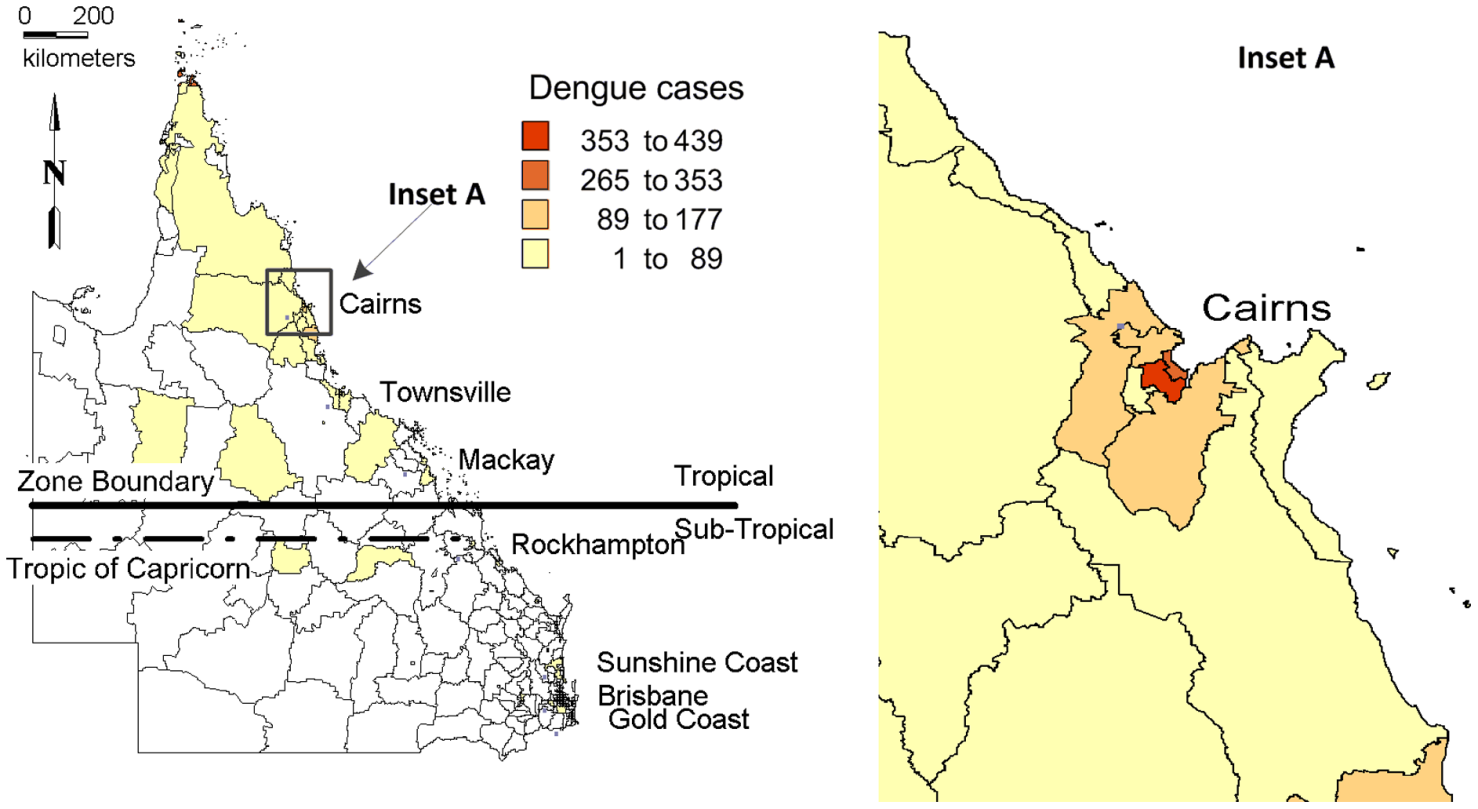
Study area showing the spatial distribution of dengue cases in northern Queensland, Australia, 1993–2012.

Queensland is the third largest state by population size in Australia (after New South Wales and Victoria), occupying a total area of 1,723,936 km^2^ with a total population of 4.56 million people (20% of Australia's total population) and is the fastest growing state with 23.9% of population growth in Australia to June 2012 [17]. Northern Queensland, a tropical region which is 100 km north of the Tropic of Capricorn (Figure 1) is selected as the study area as it had the largest total number of recorded notifications (n = 2,273) in Queensland, and the largest compared any other Australian state and territory, during the period 2005–2012 [18]. Northern Queensland has a tropical climate, with average temperatures in summer ranging from 24–33 degrees Celsius, and in winter 14–26.

### Data collection

#### Dengue data

Dengue is a notifiable disease and all positive cases are required to be reported by laboratories to the state government (Queensland Health), by the Public Health Act 2005. These records are archived by the Data Custodians, Communicable Disease Branch (CDB) unit in Queensland Health under the National Notifiable Disease Surveillance System (NNDSS). The NNDSS was established in 1990 under the auspices of the Communicable Diseases Network Australia. Vector-borne diseases notified to the NNDSS include mosquito-borne diseases caused by alphaviruses, such as Ross River virus (RRV), Barmah Forest virus (BFV) and the flavivirus, Dengue.

In Australia, dengue outbreaks are a combination of locally-acquired and overseas- or imported-cases. According to government of Queensland Health, a dengue outbreak is declared when there is one or more locally-acquired dengue cases are confirmed. An overseas- or imported-case is defined as someone who is infected with dengue overseas (i.e., viraemic traveller) and arrives Australia with the virus in their blood [19]. A locally-acquired case is defined as when a local dengue mosquito bites this overseas dengue infected person and it passes the virus on to other people by biting them [19].

We obtained computerised and anonymous notification data (data that does not contain any identifiers such as name, street and house number or Medicare number or other medical insurance number) on locally-acquired dengue cases from January 1993 to June 2012 (approximately 20 years) for the study area from the CDB, Queensland Health. Dengue data included date of notification, age group (e.g., <1, 1–4, 5–9, 10–14 etc.), gender, post code of residence and statistical local area (SLA) (census unit) name. SLA is an Australian Standard Geographical Classification (ASGC) defined area which consists of one or more collection districts (the smallest geographical unit) in Australian census. Therefore, we have analysed the data based on age group.

#### Population data

Population data for the SLAs for the national census years 2001, 2006 and 2011 were obtained from the Australian Bureau of Statistics. For the remaining years during 1993 to 2012, the annual population data were estimated based on linear interpolation. We have adjusted SLA boundaries to match earlier censuses.

The study was approved by the Data Custodians, Human Research Ethics Committee under Chapter 6, Part 4, Section 280 of the Public Health Act 2005, CDB of Queensland Health and following the ethical considerations of the Research Ethics Unit, Queensland University of Technology (Number: 1100001110).

### Statistical analyses

The study period was divided into four time periods, with five years in each time period for the ease of the analysis and to visualise the spatial and temporal patterns more clearly and precisely: Period 1: 1993–1997, Period 2: 1998–2002, Period 3: 2003–2007 and Period 4: 2008–2012. Population data for each period were attached to each SLA in the maps and these were used as the denominator in the computation of incidence rates. Period-wise distribution maps were produced on dengue cases and incidence rates by SLA. MapInfo Professional (version 11) incorporated with Vertical Mapper (version 3.7) was used to produce the final outputs as tables and maps.

### Spatial and temporal analyses

To investigate the spatial and temporal patterns of dengue disease and to determine the risk of dengue disease, monthly incidence rates were calculated at both SLA and state levels. Incidence rates for each age group and gender were also calculated from the total number of dengue cases notified in each age group for each SLA in different time periods, divided by the respective total person-years and then multiplied by 100,000. These incidence rates were expressed as: total number of dengue cases/total population*100,000. Differences between age- and gender-specific incidence rates were tested using chi-square analyses (SPSS version 21).

Age- and gender-standardised incidence rates (SIRs) were calculated for each SLA, using the direct method (based on Queensland population as a “reference”), adjusted for differences in the age and gender distribution. For example, dengue was high among the 25–29 year old age group so a SLA with a higher proportion of this age group would have a higher overall incidence rate of dengue. The equation for calculating SIR is: 
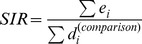
 (expressed per 100,000 people)

Where 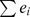 is the total number of expected cases generated using the reference population rates for each SLA; 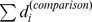 is the total population in the comparison group.

Further, the SIR estimates were mapped to identify the spatial differences. A significant difference between the observed and expected number of cases is indicated if the confidence interval (CI) does not contain zero. Differences between observed and expected number of cases for age and gender were tested using chi-square analyses.

#### Spatial analysis

We conducted spatial analysis comprising three components: 1) spatial autocorrelations, 2) semi-variogram models and 3) interpolations of SIR values (kriging).

#### Spatial autocorrelations

The global Moran's *I* test statistic was used to assess the presence of significant spatial autocorrelation of dengue overall incidence rates in each of the four time periods of 1993–1997, 1998–2002, 2003–2007 and 2008–2012. Moran's *I* ranges from −1 to 1: a value close to 0 indicates spatial randomness while a positive value indicates positive spatial autocorrelation, and a negative one indicates a negative autocorrelation, that is clustering of observations, and similarly a negative value indicates negative spatial autocorrelation, that is, repulsive behaviour between observations. Statistical significance was tested using randomisation based on 999 permutations [20]. The neighbourhood matrix used for the computation of spatial autocorrelation statistics, was based on Queen contiguity and Euclidean distance [20].

#### Semi-variogram analysis

We used semi-variogram modelling analysis to explore the spatial structure and spatial autocorrelation of SIRs of dengue and age. The semi-variogram is a graphical representation of the variation among observations as the distance between the observations increases. If the variation, measured in terms of semi-variance, is distinctly small for low values of lag distance, it is considered as an indication of positive spatial autocorrelation, i.e., values at short distance from each other are more alike than those at larger distances. The best-fit semi-variogram model was identified by using Vertical Mapper within MapInfo Professional to identify the smallest differences in the model [21].

#### Kriging interpolation

Since the semi-variogram illustrates the spatial dependency between the observed measurements as a function of the distance between them, it allows us to estimate the SIR value of dengue at any point from the observed data. This interpolation was based on the best-fit semi-variogram using a kriging method [22]–[24] with inverse distance weighting [25]. Inverse distance weighting interpolation has been employed in other analyses of mosquito-borne diseases [26]–[28]. The kriged SIR values were obtained using the interpolation method in Vertical Mapper within MapInfo Professional and mapped to better visualise the distribution of spatially related patches of dengue.

#### Temporal analysis

To examine temporal patterns, epidemic curves were produced by calculating the annual incidence rate of dengue (annual dengue cases for each year divided by total population for each year * 100,000 people) and monthly cases of dengue during the period 1993–2012. Monthly differences between incidence rates for the study period were tested using Chi-square test. The statistical significance was set at <0.05.

## Results

### Descriptive statistics

Table 1 shows summary descriptive statistics for the dengue cases for the four time periods across SLAs in northern Queensland, Australia. Overall, the average number of dengue cases was 6.82 cases per year in northern Queensland. The SLAs notified with dengue cases varied over the four time periods. There were 13 SLAs with dengue cases (n = 34) in 1993–1997, 11 SLAs in 1998–2002 (n = 130 cases), 58 SLAs in 2003–2007 (n = 1042 cases) and 58 SLAs in 2008–2012 (n = 1192 cases). In all these periods, seasonal differences were observed between the dengue cases (Table 1). During 1998–2002 and 2008 to 2012, higher number of dengue cases (n = 86 and 773, respectively) were documented in summer whereas during 1993–1997 and 2003 to 2007, higher number of dengue cases (n = 29 and 625, respectively) were recorded in autumn.

**Table 1:** Descriptive statistics of dengue cases among SLAs in northern Queensland, Australia, 1993–2012.

Indicators	1993–1997	1998–2002	2003–2007	2008–2012	Total
No. of SLAs positive	13	11	58	58	140
Total cases in all SLAs	34	130	1042	1192	2398
In summer	3	86	233	773	1095
In autumn	29	43	625	350	1047
In winter	1	0	50	17	68
In spring	1	1	134	52	188

**Figure 2:**
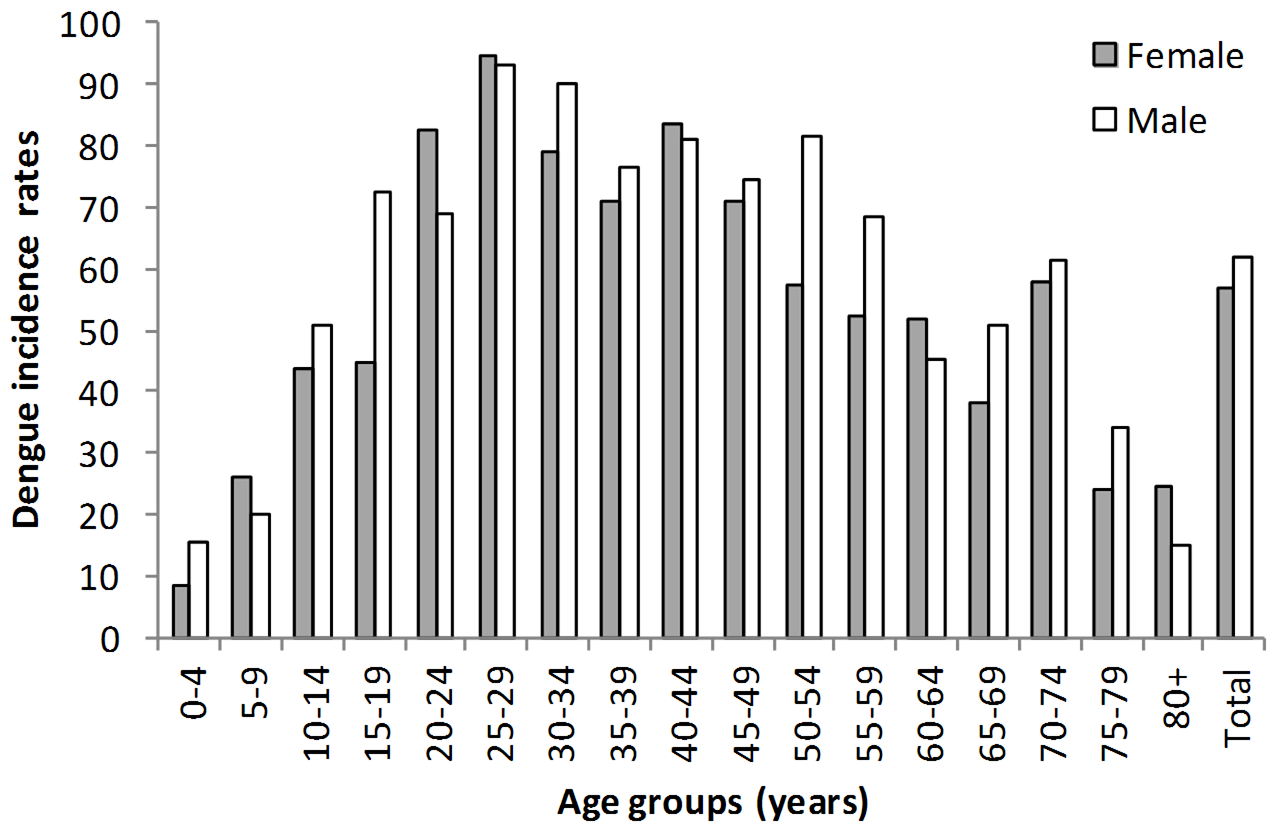
Dengue incidence rates by age and gender in northern Queensland, Australia, 1993–2012.

Figure 2 shows the age- and gender-specific distribution of dengue incidence rates (per 100,000 people) during 1993–2012 for northern Queensland. The age and gender distributions are comparable with the last national population census data in 2011 [29]. The median age of the dengue cases was 44 years (range <1–80 above years). The incidence rate increased steadily with increasing age (children aged 5–9 years to those aged 50–54 years). Females in the younger (20–25 year) age groups had slightly higher incidence rates than males in the same age groups whereas in all other age groups males had slightly higher incidence rates compared with females. This difference was statistically significant (χ^2^ = 15.17, d.f. = 1, p<0.01). Differences were observed among age groups, but these were not statistically significant.

### Temporal analysis

**Figure 3:**
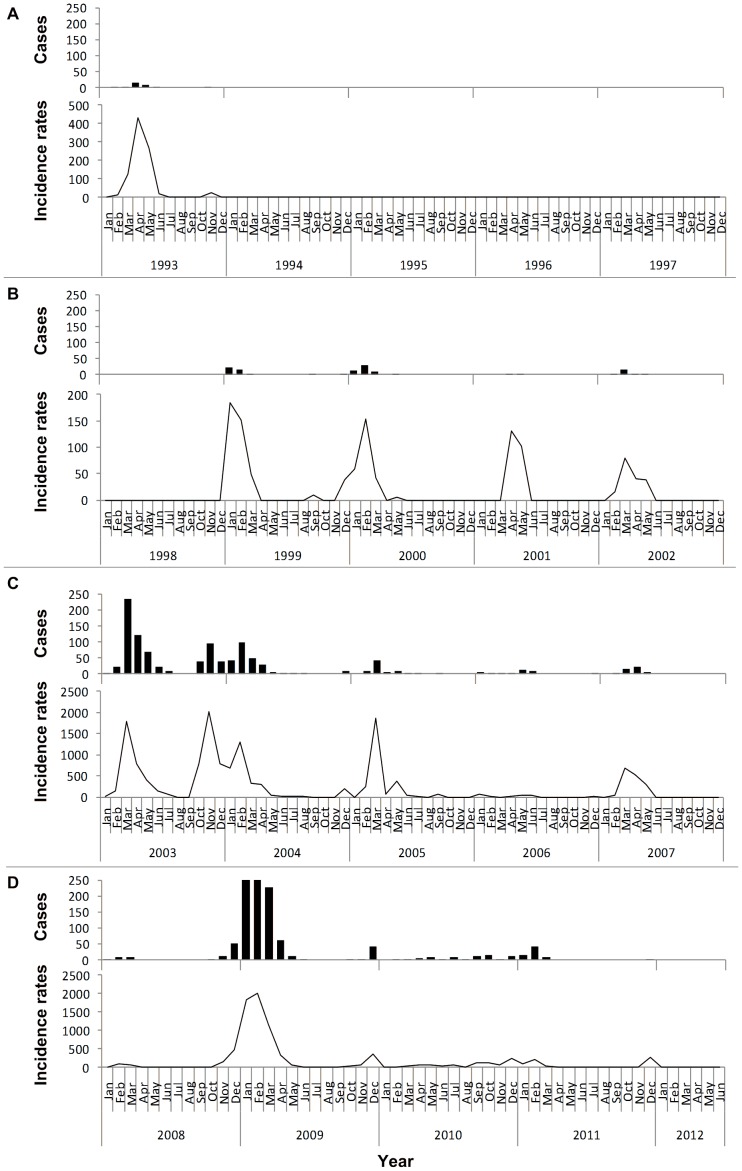
Temporal distribution of dengue cases (depicting bar) and incidence rates (depicting line) for different time periods (A: 1993–1997, B: 1998–2002, C: 2003–2007 and D: 2008–2012).

Figure 3 displays the temporal epidemic patterns of dengue cases and incidence rates for four time periods in northern Queensland, including four major outbreaks in 2003, 2004, 2005 and 2009. The annual incidence rates fluctuated from 5.65 per 100,000 people (in 2000) to 82.84 per 100,000 people (in 2005).

**Figure 4:**
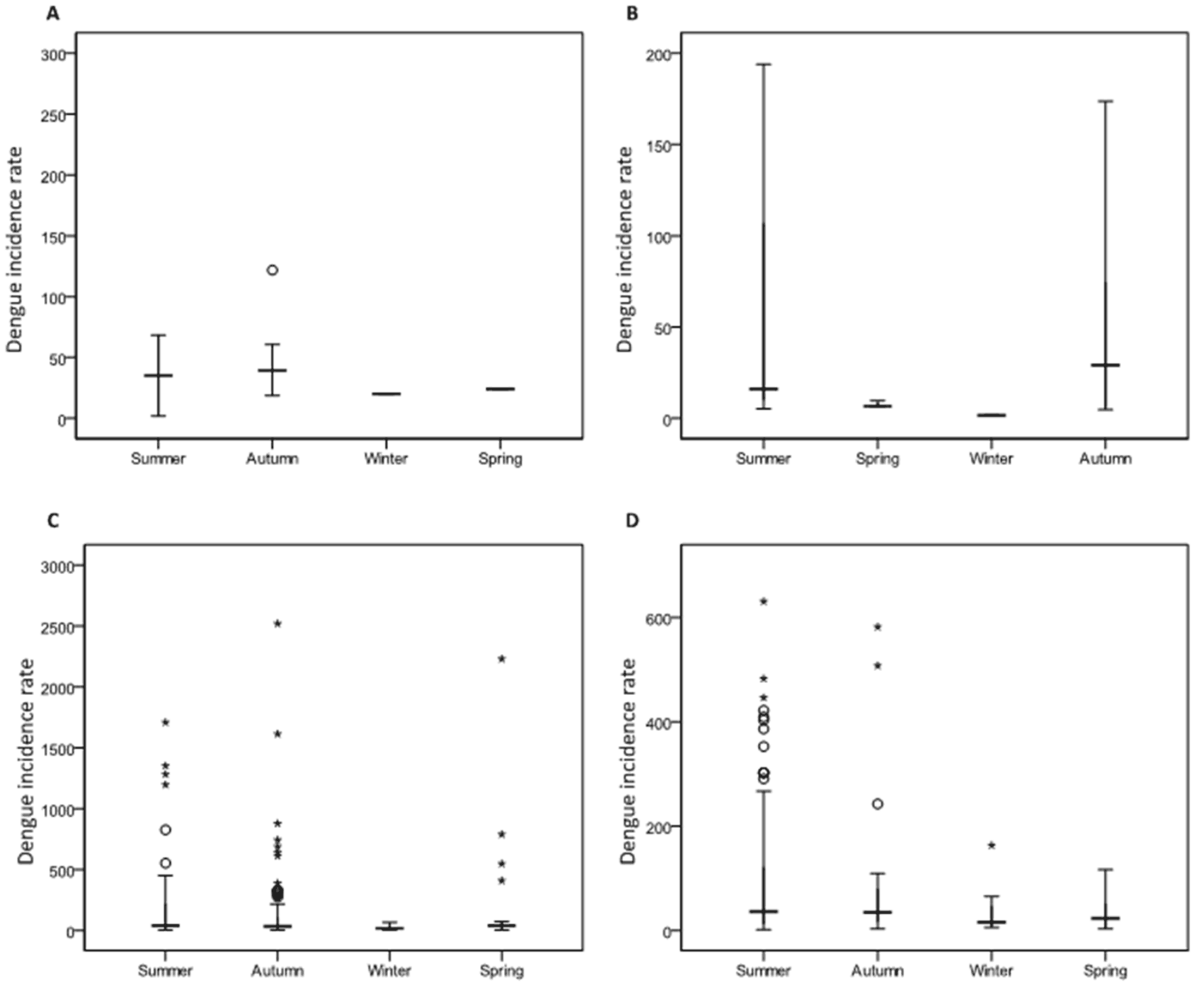
Box-plots showing the seasonal distribution of dengue cases for the different periods: A: 1993–1997, B: 1998–2002, C: 2003–2007 and D: 2008–2012.

The distribution of dengue cases and incidence rates was highly seasonally sensitive over the whole study period and in each of the four time periods. The incidence rates of dengue for the years 1993 to 2012 in northern Queensland indicates a strong seasonal pattern (χ^2^ = 8.357, d.f. = 1, p<0.01), with a peak in Autumn (i.e., March) and reduction in Winter (i.e., August), with striking differences (Fig 4) among the four time periods. This supports the observed differences in cases in Table 1.

### Spatial analysis

**Figure 5:**
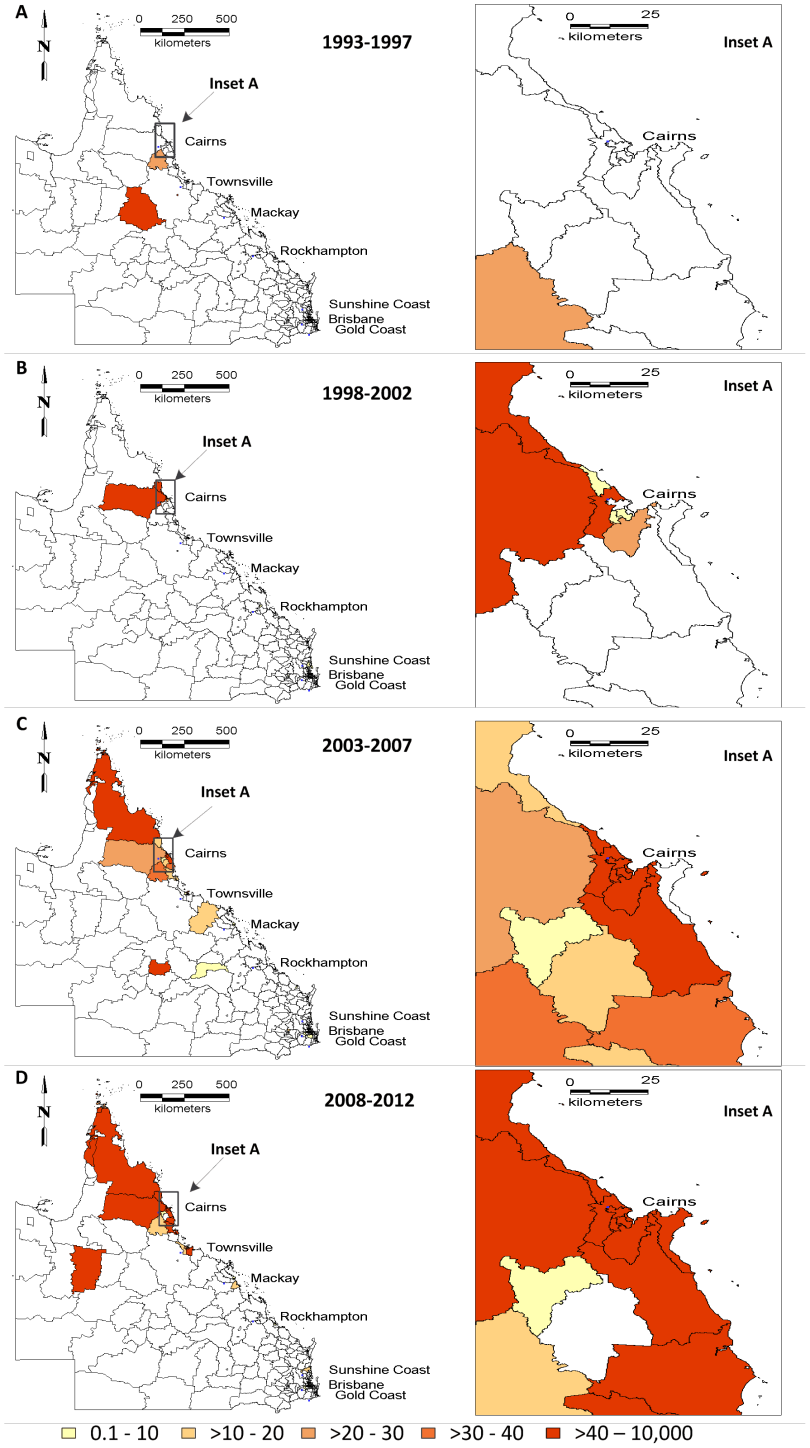
Maps showing the dengue incidence rates by SLA over different periods (A: 1993–1997, B: 1998–2002, C: 2003–2007 and D: 2008–2012).

Figure 5 depicts the spatial patterns of dengue incidence rates in the four time periods during 1993–2012. Overall and in each period, the far north Queensland regions had the highest incidence rates: SLAs with highest incidence rates were Torres followed by Cairns City (central suburbs) for the years 2008 to 2012. Other relatively large incidence rates were reported in south Townsville and Cairns central suburbs. In all the time periods, incidence rates were high in Torres, Cairns City (central suburbs), Cairns - Mt Whitfield, South Townsville, Currajong, North Ward - Castle Hill, South Flinders and Rowes Bay - Belgian Gardens. Overall, dengue appears to have spread dramatically from Cairns regions to north Queensland regions during 1993–2012. In general, dengue occurred for 15 consecutive years from 1998 primarily in the same SLAs along the coastal northern Queensland regions and gradually expanded into newer SLAs, with the highest number of cases (n = 956) reported in 2009 during January to April.

#### Spatial autocorrelation

Table 2 shows the results of the calculation of global autocorrelation statistics for dengue cases for the four time periods in northern Queensland. The results of the global Moran's *I* tests for dengue incidence rates for all four periods are statistically significant and indicate spatial heterogeneity. There was an increase in spatial autocorrelation over the period 2003–2007, reaching the highest value during 2008–2012.

**Table 2:** Moran's *I* values for the dengue incidence rates in northern Queensland, Australia, 1993–2012.

Period	Moran's *I* value	p-value
1993–1997	0.0116	0.03
1998–2002	0.0019	0.001
2003–2007	0.1362	0.001
2008–2012	0.4639	0.001

#### Standardised incidence rates

**Figure 6:**
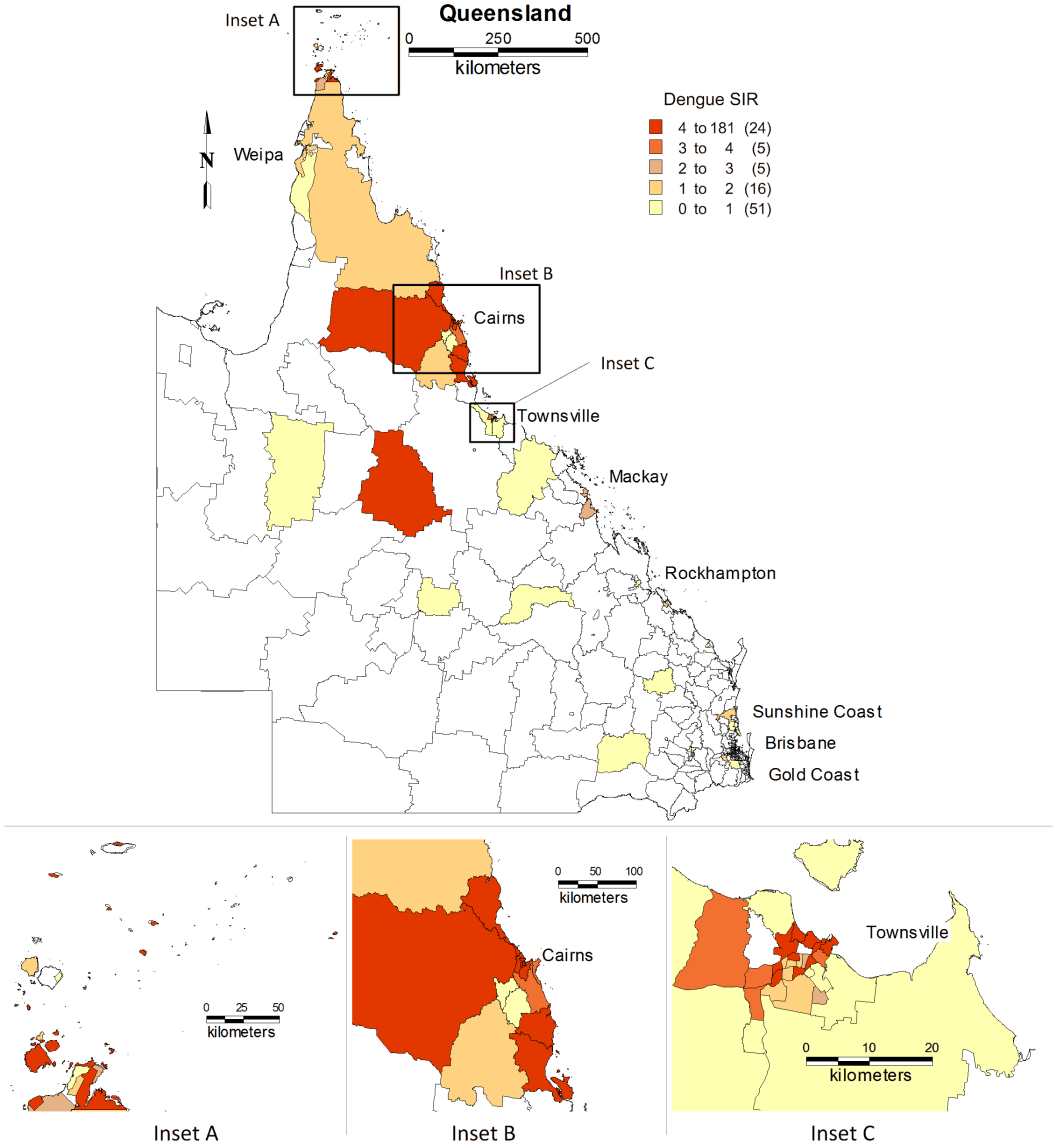
Map showing the standardised incidence rates of dengue by SLA in northern Queensland, Australia, 1993–2012.

Standardised incidence rates (SIRs) of dengue for each SLA in northern Queensland over the the study period were calculated and mapped (Fig 6). Geographically, the largest SIRs were observed in the coastal areas; with the peak SIR of 180/100,000 for Cairns City (central suburbs) while the average SIR in northern Queensland was 2.06/100,000.

#### Semi-variogram analysis and kriging

**Figure 7:**
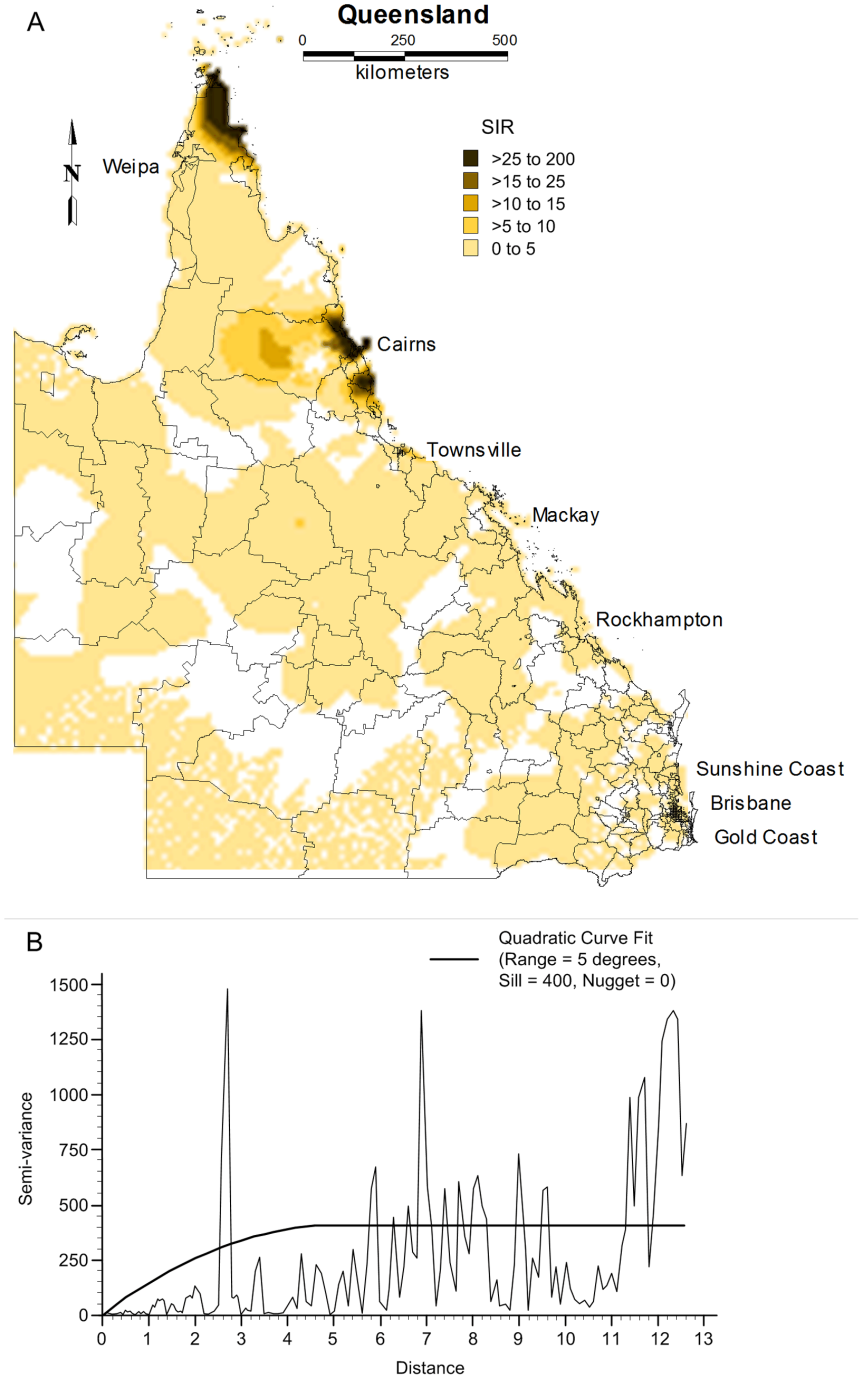
The smoothed map of standardised incidence rates of dengue using kriging (Panel A) and a semi-variogram model (Panel B).

Spatial dependence of dengue SIRs was evaluated using semi-variograms. A quadratic model was fitted to the semi-variogram using a sill and nugget of 400 and 0, respectively and a range of 5 degrees (Fig 7). This best-fit semi-variogram model was then used in the kriging procedure to map the SIRs. The map of the kriged SIR is shown in Figure 7. This provides visual confirmation that the pattern of SIRs of dengue disease is not homogeneous across the northern Queensland.

## Discussion

This study reveals the spatial and temporal characteristics of dengue in northern Queensland using GIS tools and geostatistical analytical techniques. These methods have been applied to other vector-borne infectious diseases to study the distribution patterns of the disease, to identify the high-risk areas or hot spots, and to determine the risk factors for the transmission of the disease [26], [30]–[35]. Locally-acquired dengue cases only occurred in northern Queensland where the vector was common and where the virus was commonly introduced by viraemic travellers [36]. Our results indicate that dengue incidence rates had an uneven spatial distribution in northern Queensland and thus the disease was spatially heterogeneous. In addition, the GIS maps clearly revealed spatial expansion of dengue transmission in northern Queensland over recent years (i.e., 2008–2012). For example, dengue transmission has increasingly spread from Cairns regions to a wider area.

Temporally, significant differences were noticeable across different time periods in the study area. Our findings demonstrate that the annual incidence rates fluctuated considerably, with the two largest peaks in 2003 and 2009 (see Fig 2). It is important to understand the influence of annual seasons on transmission cycles. Our results also show that there has been an increasing trend in the incidence rates of dengue during the study period. The overall temporal pattern of dengue incidence in northern Queensland indicates two general peaks per annum: the first short peak in summer (i.e. extending for two months in November and December), and a second long peak in autumn (i.e. four months from January to April). These two peaks may be preceded by peaks of mosquito densities in December and May, following the normal typical wet season. Our findings strongly support previous studies that have suggested an increase in mosquito population in summer, with a lagged impact on the seasonal variation of dengue [37], [38]. However, whether this corresponds to the peaks of tourists who might introduce the virus is still unknown.

The study results indicate that dengue outbreaks usually occur during the rainy season (i.e., November to April) in northern tropical Queensland, which generally has a lower temperature by a few degrees and higher humidity than during the hot dry season. These small environmental fluctuations may possibly increase the female mosquito longevity and survival, thus increase the likelihood of dengue transmission. Our findings strongly support previous studies that have reported a strong seasonal pattern of dengue [2], [37], [39].

The spatial and temporal differences may due partly to occurrence of epidemics due to introduced cases, mosquito distribution, local changes in climate and people's culture and behaviour towards personal protection, human adaptation to forecasted drought (e.g., water storage facilities) and housing conditions and life-style activities [2], [40] and partly to local vector control programs such as dengue mosquito management plan and dengue alert response team activities [4]. Thus, further research is needed to investigate the social mobilisation, entomological surveillance, vector biology, dengue viruses, local people's behaviour and the local climatic factors on the transmission of dengue in the northern Queensland region.

In this study, locally-transmitted dengue was reported in a small number of SLAs across the study area. The study results reveal that quite a few numbers of SLAs had the highest incidence rates and SIRs (see Figs 4 & 7). This may be due to the several conditions that favour vector, density, distribution, survival and longevity [2], [39], [41]. A combination of flooding and heavy rainfall has resulted in dengue epidemics across Australia [2], [4], [40]. In addition, local factors such as the climate and socio-ecological conditions may be more suitable for the local mosquitoes and thus the transmission of dengue. Therefore, it is evident that further studies on the environmental and socio-ecological factors on dengue mosquitoes and dengue virus would assist in identifying the reasons behind this phenomenal variation.

In this study, dengue incidence occurred in all age groups, but was highest among males and females of age group 25–29 (see Fig 3). The reasons for the differences in incidence rates among age and gender groups are unknown, but may include different exposure rates or other behavioural risk factors such as increased internal (domestic) travel, immigration, work and leisure related activities [29], however, this kind of information is unavailable in this study. The possible reasons could be increased levels of time spent outdoors for recreation (e.g., fishing and walking) and leisure (e.g., sport and exercise) activities and internal (within the state) travel due to work and family commitments. Clearly, the relationships between the incidence rate of dengue, age and gender needs to be understood in order to better manage and reduce incidence spikes in certain age groups.

Epidemiologists normally use the ratio of case numbers at a particular time to past case occurrences using the mean or median [42]. However, since dengue cases vary from one place to another, the spatial and temporal component must also be taken into consideration. In disease surveillance and public health surveys, spatial and temporal patterns are one of the most important components influencing the distribution of diseases. Although spatial analytical techniques rarely provide reasons for the occurrences of spatial patterns, they do identify the geographical locations of the occurrence of spatial pattern. Within this realm, it provides a useful means to hypothesise about factors that may influence health outcomes or to identify spatial issues that need to be further investigated [43]. The evaluation of spatial distributions as a measure of disease risk may provide etiological insights [44]. Regular time series models are difficult to fit to our data, given the epidemic and irregular seasonal patterns of dengue incidence. Thus, we haven't pursued these models in this study.

In this study, the global Moran's *I* statistic is used to measure the degree of spatial autocorrelation and map the geographic patterns of the areal units. To appropriately use dengue notification data aggregated according to SLAs, it is important to choose the spatial autocorrelation technique for the specification of local neighbourhoods. This is defined by the spatial weights matrix. In general, the spatial autocorrelation may be the strongest between the nearest neighbours. As the neighbourhoods increase in number, this autocorrelation weakens. However, a recognised guide for choosing a proper spatial weight matrix has not yet been developed. In this study, an appropriate spatial weight matrix was chosen after a comparison of the connectivity distributions of neighbours obtained with the distance-based contiguity and the first order Queen's contiguity methods [20].

Spatial autocorrelation and semi-variogram analysis are valuable tools to study spatial patterns over time. The semi-variogram estimators used in this paper directly account for population size, attenuating the influence of less reliable rates recorded in sparsely populated areas. Maps created from kriging interpolation revealed that dengue was spatially and temporally distributed. Further studies of local environmental and socio-environmental factors that operate at fine spatial scales are crucial for improving the understanding of the spatial and temporal patterns of dengue. Moreover, further investigation is warranted to understand the effect of climatic and topographic factors on dengue local transmission in the study area.

Vector-borne infectious diseases such as dengue present complex and dynamic transmission patterns, which include vector related factors such as type, density, distribution, breeding places and human related factors such as population density, behaviour and immunity, and virus related factors such as circulating multiple virus serotypes DENV 1 to DENV 4, and environmental factors such as temperature and rainfall [1], [11], [45]. Several dengue outbreaks by different serotypes may occur in the same population, and there is a large range of factors in intra-urban areas which may favour the maintenance of potential breeding sites of mosquitoes.

We acknowledge that there could be issues in monitoring and reporting dengue locally-acquired notification data. For this study, the clinically proven cases on dengue were provided by Queensland Department of Health. Dengue is one of the notifiable diseases in Australia, and is required to be reported to the local health, by law. Data reliability issues for mosquito-borne diseases have previously been discussed by Russell [46]. Underreporting is also likely to occur when people infected with dengue do not seek medical attention. For example, it is likely that there was considerable underreporting in 1992 and 1993 but we have evidence to suggest that in more recent epidemics the publicly notified cases are not a gross underestimate of the total number of cases. Nevertheless, these issues cannot entirely account for the geographic distribution of dengue across northern Queensland.

This study has three major strengths. Firstly, this is the first study to examine the geographic variation of dengue across geo-political borders (i.e., SLAs) in northern Queensland using GIS tools and geostatistical techniques. This study is an impetus for future investigations on the spatial and temporal risk factor analysis of dengue including multiple factors on environmental, entomological, ecological and socio-demographic variables. Secondly, the results of this study demonstrate that GIS mapping techniques may be used as a tool to quickly display information and generate maps to highlight dengue risk-prone areas for developing strategies towards dengue management. The maps could be used by policy-decision makers to suggest particular geographical localities or communities where further investigation should be focused, to identify whether increased disease surveillance measures or possible control activities are warranted. Moreover, the corresponding statistical analyses can limit over-reaction or unwarranted action based on purely visual assessment, for example unsubstantiated identification of so-called ‘clusters’ of cases and putative source identification. Finally, dengue data used in this study are quite comprehensive, covering northern Queensland for approximately 20 years.

The study has also two key limitations. First, the quality of the dengue surveillance system may vary with place and time as the awareness of dengue among medical professionals and public may have increased over recent years. However, spatiotemporal variation of dengue infers that the dengue distribution is unlikely to be entirely accounted for by a detection/surveillance. Second, the exact place/location (i.e., residential address) where dengue cases were notified may vary from those where they were infected/acquired (i.e., exact place/location), particularly during holiday periods. However, all the cases involved in this study were locally-acquired and were not imported.

The maps produced in this study would provide useful information to health authorities and could assist in focusing and implementing control and preventive activities to monitor and control the incidence of dengue precisely and effectively, especially in the event when there is no report on dengue cases. The study suggests that local surveillance teams should be vigilant at all times, particularly after the wet season, not only in the epidemic period, as dengue mosquitoes live with the human population. Furthermore, this study provides a new dimension to the health authorities in northern Queensland, specifically in the potential of using GIS applications to develop locally appropriate and eco-environmentally friendly strategies for the implementation of preventive and control activities, not only for dengue, but also for other vector-borne diseases in Queensland.

In conclusion, this study has revealed that the spatio-temporal patterns of dengue differ significantly in northern Queensland and the study has highlighted that there are different transmission patterns in SLAs between regions. This study has also concluded that the spatial distribution of dengue appears to have expanded over recent years. This is based on the results (see Tables 1 & 2) and on the observation that dengue has spread from Cairns regions to a wider area during 1993–2012. Autocorrelation function can be beneficial for public health officials or policy-developers to visualise and understand the distribution and trends of diffusion patterns of dengue and to prepare warnings over high-risk areas only rather than for a whole state or a whole region. This may save time and cost and make public health authorities efforts more efficient.

Future research should focus on the spectrum of dengue risk factors and the prediction of future dengue transmission which are necessary to improve the effectiveness and efficiency of dengue local prevention and control programs.
